# Workplace exposure to diesel and gasoline engine exhausts and the risk of colorectal cancer in Canadian men

**DOI:** 10.1186/s12940-016-0088-1

**Published:** 2016-01-14

**Authors:** Linda Kachuri, Paul J. Villeneuve, Marie-Élise Parent, Kenneth C. Johnson, the Canadian Cancer Registries Epidemiology Research Group, Shelley A. Harris

**Affiliations:** Dalla Lana School of Public Health, University of Toronto, 155 College Street, 6th Floor, Toronto, ON M5T 3 M7 Canada; Occupational Cancer Research Centre, Cancer Care Ontario, 525 University Avenue, 3rd Floor, Toronto, ON M5G 2 L3 Canada; Prevention and Cancer Control, Cancer Care Ontario, 620 University Ave, Toronto, ON M5G 2 L7 Canada; CHAIM Research Centre, Carleton University, 5435 Herzberg Laboratories, 1125 Colonel By Drive, Ottawa, ON K1S 5B6 Canada; INRS-Institut Armand-Frappier, Institut national de la recherche scientifique, University of Quebec, 531 boul. des Prairies, Édifice 12, Laval, QC H7V 1B7 Canada; Department of Epidemiology and Community Medicine, University of Ottawa, 451 Smyth Rd, Ottawa, ON K1H 8 M5 Canada

**Keywords:** Diesel emissions, Gasoline emissions, Colon cancer, Rectal cancer, Colorectal cancer, Occupational cancer, Case–control study

## Abstract

**Background:**

The International Agency for Research on Cancer (IARC) classified diesel exhaust as carcinogenic to humans (Group 1) and gasoline exhaust as a possible carcinogen (Group 2B) based studies of lung cancer, however the evidence for other sites is limited. We addressed this question by investigating exposure to diesel and gasoline emissions with respect to risk of colorectal cancer in men.

**Methods:**

We used data from a population-based case–control study with incident cases of colon (*n* = 931) and rectal (*n* = 840) cancer and 1360 controls from 7 Canadian provinces conducted in 1994–1997. Lifetime occupational history and information on other risk factors was collected. Occupational hygienists, blinded to case–control status, assigned exposures to each job for 3 dimensions: concentration, frequency, and reliability. Logistic regression was used to estimate odds ratios (OR) and their 95 % confidence intervals (CI), adjusted for age, province, use of proxy respondents, smoking, body-mass index, physical activity, intake of alcohol, processed meats, and occupational exposure to asbestos and aromatic amines.

**Results:**

Among CRC cases, 638 (36 %) were exposed to diesel and 814 (46 %) were exposed to gasoline emissions. Relative to the unexposed, elevated risks were observed among subjects ever exposed to high concentration levels of diesel emissions for colorectal cancer (OR = 1.65, 95 % CI = 0.98–2.80) and rectal cancer (OR = 1.98, 95 % CI = 1.09–3.60), but not colon cancer. Prolonged (>10 years) exposure at high concentrations was also associated with high risks of rectal cancer (OR = 2.33 95 % CI = 0.94–5.78; p-trend = 0.02). No statistically significant associations were observed for gasoline emissions.

**Conclusions:**

Our findings suggest that sustained high-level exposure diesel emissions may increase the risk of rectal cancer.

**Electronic supplementary material:**

The online version of this article (doi:10.1186/s12940-016-0088-1) contains supplementary material, which is available to authorized users.

## Background

Motor vehicle exhaust emissions are ubiquitous, with exposure occurring through indoor and outdoor air, as well as in many occupational settings. An estimated 781,000 workers (92% of employed males), or 4.6 % of the working Canadian population, are exposed to diesel engine exhaust [1, 2]. The two largest exposed occupational groups are truck drivers and heavy equipment operators [1, 2]. Diesel and gasoline are the most widely used fuels in combustion engines, and their emissions are comprised of a complex mixture of chemicals, including polycyclic aromatic hydrocarbons (PAHs) and nitroarenes, carbon monoxide, and volatile organic compounds, such as benzene and formaldehyde [3, 4]. While similar particles are emitted from both gasoline and diesel powered engines, the distribution and surface properties of the particles are different, which suggests potential differences in the health effects associated with these exposures [5].

The International Agency for Research on Cancer (IARC) recently classified diesel exhaust as carcinogenic to humans (Group 1), based on studies of lung cancer, an update from the 1988 classification of probably carcinogenic to humans (Group 2A) [3]. However, the evidence for other cancers remains limited. Gasoline was classified as a possible human carcinogen (Group 2B) by IARC in 1989 and again in 2012, based on inadequate evidence in humans and sufficient evidence in experimental animals [3]. Although in most developed countries exposures to gasoline emissions have surpassed those from diesel, few studies have examined its effects on cancer risk, especially for sites other than the lung.

Colorectal cancer (CRC), affecting the colon and/or the rectal areas, is a major contributor to the worldwide cancer burden [6]. CRC has been linked to several modifiable risk factors including obesity, physical inactivity, consumption of red and processed meat and smoking [7–9]. In addition to physical activity, a potential role for other occupational risk factors has been suggested [10, 11]. To date, few studies have investigated the relationship between exposure to diesel and gasoline emissions and CRC risk, but they did report modest positive associations [12–14]. However, only two case–control studies used a systematic and validated approach to exposure assessment [12, 13], and many of these studies lacked detailed information on several major lifestyle-related CRC risk factors, such as body-mass index (BMI), diet and physical activity.

Currently, the overall evidence linking diesel and gasoline emissions to CRC is considered to be weak [3]. Further investigating the carcinogenic potential of these prevalent exposures has been identified as a high priority by IARC [15]. To our knowledge, this is the largest population-based case–control study of the effects of diesel and gasoline emissions on the risk of colon and rectal cancer, with a comprehensive exposure assessment strategy, along with detailed information on important confounders. This work addresses an important gap in our understanding of the extensive health effects of exposure to diesel and gasoline emissions, and has implications for preventive efforts.

## Methods

### Study population

The data used in this analysis are from the colon and rectal cancer case–control components of the Canadian National Enhanced Cancer Surveillance System (NECSS), a collaborative effort between Health Canada and the cancer registries in 8 Canadian provinces. The NECSS collected data from a population-based sample of 21022 cases of 19 different cancers and 5039 controls between 1994 and 1997. Data for rectal and colon cases were not collected in Ontario, therefore this analysis is based on male participants from 7 provinces. Cancer-free controls were recruited using a random sample of the provincial population obtained from health insurance plans in 5 provinces (Prince Edward Island, Nova Scotia, Manitoba, Saskatchewan and British Columbia) and random-digit dialing in Newfoundland and Alberta [16]. Frequency-matching to the overall case grouping (19 cancer sites) was used to select controls for every case within each sex and 5-year age group for any cancer site.

Of the total cases ascertained, physician consent was obtained for 89 % of rectal (*n* = 1169) and 85 % (*n* = 1375) colon cases. Deceased cases were excluded (76 rectal; 138 colon), as well as patients whose physician refused consent (71 rectal; 98 colon). The NECSS collected detailed information for a number of modifiable cancer risk factors, including: sociodemographic characteristics, anthropometry, diet, smoking, alcohol use, exposure to environmental tobacco smoke, and physical activity. Individuals were also asked to provide details on their lifetime residential and occupational history. Information on diet from 2 years prior to the interview was collected using a modified 60-item food-frequency diet questionnaire based on instruments that have been extensively validated [17, 18]. Completed questionnaire were returned by 830 rectal and 959 male colon cases, representing 71 and 70 % of those contacted, respectively. Questionnaires were mailed to 4270 men who were identified as eligible controls. Approximately 7 % (*n* = 287) were returned due to an incorrect address. Completed questionnaires were received from 2547 male controls, representing 59.6 % of those ascertained and 64.0 % of those contacted.

The study sample was restricted to men because we expected few women to be occupationally exposed to diesel and gasoline emissions during the relevant exposure periods (between the 1960s–1990s). We excluded individuals under the age of 40, and those who had not worked for at least one year, since individuals with shorter employment histories would have had fewer opportunities for exposure, and their CRC diagnoses would be more likely attributable to genetic conditions, such as familial adenomatous polyposis and hereditary non-polyposis colorectal cancer, which confer lifetime risks as high as 70–80 % [19, 20], with a mid-40s average age at diagnosis [21]. In the NECSS, 2.9 % (*n* = 28) of incident colon cancer and 2.1 % (*n* = 18) rectal cancer cases were diagnosed before the age of 40; the corresponding number of controls excluded to meet the age requirement was 282. After applying the exclusion criteria there remained a total of 931 colon and 840 rectal cancer cases, and 1360 controls.

### Assignment of occupational exposures

Participants were asked to provide information for each job held, in Canada, for at least 12 months from the time they were 18 years old to the time of the interview. The information for each occupation included the job title, main tasks performed, type of industry, location, and period of employment. Using these job descriptions, a team of chemists and industrial hygienists, who were blinded to case or control status, carried out a comprehensive exposure assessment and determined the exposure status of each job with respect to diesel emissions, gasoline emissions, aromatic amines, asbestos and crystalline silica.

As in our previously published studies of lung cancer [22–24], the occupation and industry coding was reviewed and upgraded to the more precise 7-digit Canadian Classification and Dictionary of Occupation (CCDO) codes [25] which were used in the subsequent steps of the exposure assessment. A total of 16,009 jobs contained sufficient occupational information for the exposure assessment. Exposure metrics were not assigned to jobs with insufficient information (*n* = 199), including those missing full-time, seasonal or part-time employment status (*n* = 74). In addition, jobs were not coded for diesel or gasoline emissions exposure if they were self-reported as retirement (*n* = 87), student (*n* = 35), disability (*n* = 5), institutionalized (*n* = 1), and unemployed (*n* = 10).

The occupational exposure assessment was based on the expert-approach, where a team of industrial hygienists assigned individual exposures based on details provided for each job [26]. This approach is considered to be reliable and recognized as the reference method for such a study design [27]. It has been described in detail elsewhere [28], and has been successfully applied to other case–control studies [28, 29] and previously published analyses of NECSS data [22–24]. When assigning exposures, hygienists had access to previous NECSS expert-based codings, summarized as a job exposure matrix, which they could modify to reflect circumstances specific to each job description. Exposure coding was repeated for a random subset of 96 participants with 385 jobs to estimate the reliability of the exposure coding. Our analysis of this subset suggests excellent inter-rater agreement between exposure coders (weighted kappa = 0.81, 95 % CI: 0.78-0.85; manuscript in preparation).

The assignment of exposure to diesel and gasoline emissions was carried out across three dimensions: concentration, frequency, and reliability. Each of these variables was defined using a semi-quantitative scale: none (unexposed), low, medium, or high. Non-exposure was defined as exposure up to background levels found in the general environment. The frequency of exposure was determined based on the proportion of time in a typical workweek that the participant was exposed to a substance. Low frequency of exposure corresponded to <5 %, 5 %–30 % was classified as medium, and high frequency was defined as >30 % of work time. This metric also accounted for part-time or seasonal employment. Concentration was assessed on a relative scale with respect to established benchmarks. Low concentration was assigned to subjects with the lowest possible concentration that could still be considered above the background level of the general population. The same exposure assessment approach was also applied to a number of other exposures of interest and potential confounders, including crystalline silica, asbestos and aromatic amines.

The reliability metric was used to measure the confidence that the exposure was actually present in the job that was evaluated. Low reliability refers to “possible” exposure, medium reliability was interpreted as “probable” exposure, and high reliability corresponded to “definite” exposure. In order to reduce the potential for exposure misclassification, all exposure metrics that used in this analysis classified estimates with a low reliability score corresponding to “possible” exposure (*n* = 613, 30.4 % for diesel and *n* = 342, 19.7 % for gasoline) as unexposed.

### Exposure to diesel and gasoline emissions

The effects of occupational exposure to diesel and gasoline emissions were modeled using 5 exposure metrics: ever exposure, highest attained exposure concentration, duration of exposure, frequency of exposure, and cumulative exposure. Ever exposure was modeled as a binary variable indicating that an individual had worked in a job that was classified as exposed across any of the dimensions evaluated in the exposure assessment. The highest attained exposure concentration corresponded to the maximum concentration value assigned to jobs with an individual’s employment history. Duration of exposure corresponded to years of employment in occupations classified as exposed. Duration was modeled continuously, as well as using ordinal categories based on tertiles of exposure duration among the controls. Each set of cut-points was determined separately for diesel and gasoline emissions.

The cumulative measure of diesel and gasoline exposure was defined as:

$$ CE={\displaystyle \sum_{i=1}^k{C}_i{F}_i{D}_i} $$

where *CE* was the cumulative exposure, *i* represented the *i*^*th*^ job held, *k* was the total number of jobs held, *C* was the concentration of diesel or gasoline exposure (low, medium, high), *F* was the frequency of exposure (low, medium, high), and *D* was the duration of employment in years.

### Statistical analysis

Odds ratios (OR) and 95 % confidence intervals (CI) were estimated using unconditional logistic regression for each of the exposure metrics. Trends were also examined using logistic regression. Ordinal metrics of diesel and gasoline exposure were treated as continuous in the regression model in order to obtain an estimate of the slope and associated p-values (p-trend). Descriptive analyses were conducted and the influence of possible risk factors suggested by the literature and by previous analyses of NECSS data was investigated. These included the characteristics presented in Table 1, as well as additional nutritional factors, including consumption of red meat and intake of added fat. Exposure metrics for diesel and gasoline emissions were entered into the statistical models together in order to account for the simultaneous exposure to both of these constituents of motor vehicle exhaust. Additional occupational confounders and potential co-exposures included asbestos and aromatic amines, which were entered into the models as dichotomous variables.

**Table 1:** Selected demographic characteristics of incident colon and rectal cancer cases and controls in the Canadian National Enhanced Cancer Surveillance System 1994–97

	Colon (*n* = 931)		Rectum (*n* = 840)		Controls (*n* = 1360)		OR^a^	95 % CI	
	N	(%)	N	(%)	N	(%)			
Age groups (years)									
40 to <50	67	(7.2)	79	(9.4)	137	(10.1)			
50 to <60	174	(18.7)	179	(21.3)	239	(17.6)			
60 to <70	445	(47.9)	380	(45.2)	581	(45.7)			
70≤	244	(26.2)	202	(24.1)	403	(29.6)			
Province of residence									
Newfoundland	42	(4.5)	46	(5.5)	105	(7.7)			
Prince Edward Island	20	(2.2)	17	(2.0)	63	(4.6)			
Nova Scotia	77	(8.3)	71	(8.5)	307	(22.6)			
Manitoba	97	(10.4)	85	(10.1)	126	(9.3)			
Saskatchewan	90	(9.7)	108	(12.9)	120	(8.8)			
Alberta	283	(30.4)	184	(21.9)	265	(19.5)			
British Columbia	322	(34.6)	329	(39.2)	374	(27.5)			
Use of proxy respondents									
None (self)	579	(62.2)	485	(57.7)	903	(66.4)	1.00		
Other	352	(37.8)	355	(42.3)	457	(33.6)	1.35	(1.16,	1.57)
Education (total years)									
< 9	219	(23.5)	189	(22.5)	308	(22.7)	1.00		
9 to 11	295	(31.7)	273	(32.5)	395	(29.0)	0.99	(0.81,	1.21)
12 to 14	241	(25.9)	233	(27.7)	350	(25.7)	0.80	(0.64,	1.00)
14≤	176	(18.9)	145	(17.3)	307	(22.6)	0.61	(0.48,	0.77)
BMI (kg/m^2^)									
Normal: 18.5 to <25	251	(27.0)	254	(30.2)	490	(36.0)	1.00		
Underweight: <18.5	14	(1.5)	6	(0.7)	20	(1.5)	0.99	(0.52,	1.91)
Overweight: 25 to <30	464	(49.9)	418	(49.8)	641	(47.1)	1.44	(1.22,	1.70)
Obese: 30≤	202	(21.7)	162	(19.3)	209	(15.4)	1.89	(1.52,	2.35)
Percent change (%) from maximum lifetime weight in kg									
Q1: <2.4	287	(30.8)	233	(27.7)	280	(20.6)	1.00		
Q2: 2.4 to <4.9	226	(24.3)	202	(24.1)	359	(26.4)	0.60	(0.49,	0.74)
Q3: 4.9 to <8.8	222	(23.9)	202	(24.1)	336	(24.7)	0.68	(0.55,	0.83)
Q4: 8.8 ≤	196	(21.1)	203	(24.2)	385	(28.3)	0.56	(0.46,	0.69)
Cigarette pack-years									
Never smokers	178	(19.4)	161	(19.5)	302	(22.6)	1.00		
< 10	145	(15.8)	153	(18.6)	223	(16.7)	1.19	(0.94,	1.52)
10 to <20	173	(18.8)	136	(16.5)	233	(17.4)	1.19	(0.94,	1.50)
20 to <30	155	(16.9)	143	(17.3)	214	(16.0)	1.38	(1.08,	1.75)
30 to <40	107	(11.6)	104	(12.6)	147	(11.0)	1.43	(1.09,	1.88)
40 to <50	101	(11.0)	79	(9.6)	108	(8.1)	1.61	(1.20,	2.17)
50 to <60	25	(2.7)	16	(1.9)	53	(4.0)	0.86	(0.55,	1.36)
60≤	35	(3.8)	33	(4.0)	56	(4.2)	1.29	(0.86,	1.94)
Quartiles of alcohol intake (servings/week)									
None	259	(27.8)	227	(27.0)	451	(33.2)	1.00		
Q1: ≤2.0	168	(18.1)	145	(17.3)	253	(18.6)	1.04	(0.84,	1.29)
Q2: >2.0 to ≤6	157	(16.9)	131	(15.6)	247	(18.2)	1.03	(0.82,	1.28)
Q3: >6 to ≤14.5	186	(20.0)	152	(18.1)	216	(15.8)	1.29	(1.03,	1.61)
Q4: >14.5	161	(17.3)	185	(22.0)	193	(14.2)	1.50	(1.19,	1.87)
Quartiles of processed meat intake (servings/week)									
Q1: ≤1.4	216	(23.2)	192	(22.9)	427	(31.4)	1.00		
Q2: >1.4 to ≤3.5	224	(24.1)	232	(27.7)	368	(27.1)	1.32	(1.08,	1.61)
Q3: >3.5 to ≤6.5	245	(26.3)	212	(25.2)	267	(19.6)	1.81	(1.46,	2.23)
Q4: >6.5	246	(26.4)	204	(24.3)	298	(21.9)	1.49	(1.21,	1.83)
Quartiles of total vegetable intake (servings/week)									
Q1: ≤12.5	225	(24.2)	217	(25.8)	343	(25.2)	1.00		
Q2: >12.5 to ≤18.4	247	(26.5)	204	(24.3)	338	(24.9)	1.05	(0.86,	1.29)
Q3: >18.4 to ≤25.0	227	(24.4)	215	(25.6)	336	(24.7)	1.01	(0.83,	1.25)
Q4: >25.0	232	(24.9)	204	(24.3)	343	(25.2)	1.03	(0.83,	1.26)
Quartiles of total fruit intake (servings/week)									
Q1: ≤4.4	229	(24.6)	211	(25.1)	347	(25.5)	1.00		
Q2: >4.4 to ≤8.9	236	(25.4)	208	(24.8)	345	(25.4)	0.99	(0.80,	1.21)
Q3: >8.9 to ≤14	239	(26.7)	218	(26.0)	341	(25.1)	1.01	(0.82,	1.24)
Q4: >14	227	(24.4)	203	(24.2)	327	(24.0)	0.99	(0.80,	1.22)
Quartiles of moderate physical activity (hours/month)									
Q1: ≤3.8	222	(23.9)	204	(24.3)	355	(26.1)	1.00		
Q2: >3.8 to ≤12.2	240	(25.8)	206	(24.5)	339	(24.9)	1.00	(0.81,	1.23)
Q3: >12.2 to ≤25.3	244	(26.2)	196	(23.3)	343	(25.2)	0.95	(0.77,	1.17)
Q4: >25.3	225	(24.2)	234	(27.9)	323	(23.8)	1.05	(0.85,	1.29)
Quartiles of strenuous physical activity (hours/month)									
Q1: 0	460	(49.4)	397	(47.3)	673	(49.5)	1.00		
Q2: >0 to ≤1.2	151	(16.2)	151	(18.0)	221	(16.3)	0.97	(0.79,	1.20)
Q3: >1.2 to ≤7.3	167	(17.9)	136	(16.2)	230	(16.9)	0.96	(0.78,	1.18)
Q4: >7.3	153	(16.4)	156	(18.6)	236	(17.4)	0.89	(0.72,	1.10)
Composite index of strenuous and moderate physical activity^b^ (quartiles)									
Q1: Least active	203	(21.8)	186	(22.1)	340	(25.0)	1.00		
Q2	257	(27.6)	217	(25.8)	340	(25.0)	1.10	(0.89,	1.35)
Q3	221	(23.7)	190	(22.6)	340	(25.0)	0.94	(0.76,	1.16)
Q4: Most active	250	(26.9)	247	(29.4)	340	(25.0)	1.09	(0.88,	1.34)
Ever exposed to aromatic amines									
No	914	(98.2)	830	(98.8)	1348	(99.1)	1.00		
Yes	17	(1.8)	10	(1.2)	12	(0.9)	1.71	(0.84,	3.45)
Ever exposed to asbestos									
No	781	(83.9)	719	(85.6)	1209	(88.9)	1.00		
Yes	150	(16.1)	121	(14.4)	151	(11.1)	1.43	(1.15,	1.78)
Ever exposed to crystalline silica									
No	597	(35.8)	293	(34.9)	929	(68.3)	1.00		
Yes	334	(64.1)	547	(65.1)	431	(31.7)	1.09	(0.93,	1.28)

^a^OR estimated for colon and rectal cancers combined

^b^Composite index of physical activity was derived y summing the hours/month of moderate and strenuous activity

Adjustments were also made for age at cancer diagnosis or interview, province of residence, use of proxy respondents; BMI categories (<18.5 (underweight), 18.5 to <25.0 (normal), 25.0 to <30.0 (overweight), and ≥30 (obese)); percent (%) change from maximum lifetime BMI; cigarette pack-years (number of years smoking on average 20 cigarettes per day); consumption of processed meat; and physical activity. In order to comprehensively adjust for different types of physical activity, a composite index was created, which included hours per month of moderate and strenuous activity [30, 31]. Results for minimally adjusted models with age, province of residence, and use of proxy respondents as the only covariates are provided as Additional file 1: Table S1, Additional file 2: Table S2, Additional file 3: Table S3, Additional file 4: Table S4.

All analyses were conducted using SAS version 9.4 (Cary, N.C.).

The participating cancer registries obtained approval of the NECSS study protocol from their respective ethics review boards. The study was also approved by Research Ethics Boards at Health Canada and the University of Toronto.

## Results

A summary of socio-demographic and occupational characteristics is presented in Table 1. Compared to controls, CRC cases were older and more likely to require the use of proxy respondents. A statistically significant association emerged between increasing BMI and CRC risk. Consistent with previous studies, increased consumption of processed meat was positively associated with CRC (*p* < 0.05). However, there was no association with consumption of fruits and vegetables, or physical activity. Among the additional occupational agents that were evaluated, ever exposure to aromatic amines (OR = 1.73, 95 % CI = 0.84–3.45), asbestos (OR = 1.43, 95 % CI = 1.15–1.78), and crystalline silica (OR = 1.09, 95 % CI = 0.93–1.28) were associated with increased risk of CRC.

A total of 638 (36.0) CRC cases and 491 (36.1 %) controls were exposed to diesel emissions at some point during their occupational history (Table 2). Exposure to gasoline emissions was more prevalent than diesel emissions, with 814 (46.0) cases and 577 (57.6 %) controls exposed (Table 3). Ever exposure to either diesel (OR = 0.88, 95 % CI = 0.74–1.06) or gasoline (OR = 1.10, 95 % CI = 0.93–1.31) was not associated with statistically significant increases in the odds of CRC. Similar results were observed when colon and rectal cancers were considered separately (Tables 4 and 5).

**Table 2:** Adjusted odds ratios (OR) and corresponding 95 % confidence intervals (CI) for colorectal cancer in relation to occupational exposure to diesel emissions

Exposure Metric^a^	Cases (%)		Controls (%)		OR^b^ (95 % CI)		
Ever exposed							
No	1133	(64.0)	869	(63.9)	1.00		
Yes	638	(36.0)	491	(36.1)	0.88	(0.74,	1.06)
Highest attained exposure concentration							
Unexposed	1133	(64.0)	869	(63.9)	1.00		
Low	450	(25.4)	377	(27.7)	0.79	(0.65,	0.96)
Medium	136	(7.7)	89	(6.5)	1.00	(0.73,	1.36)
High	52	(2.9)	25	(1.8)	1.65	(0.98,	2.80)
p-trend					0.78		
Duration of exposure (years)							
Unexposed	1133	(64.5)	869	(64.4)	1.00		
> 0 to <11	208	(11.9)	157	(11.6)	0.90	(0.70,	1.16)
≥ 11 to ≤31	220	(12.5)	166	(12.3)	0.91	(0.71,	1.18)
> 31	195	(11.1)	157	(11.6)	0.80	(0.60,	1.08)
p-trend					0.11		
Duration of exposure at high concentration (years)							
Unexposed	1719	(97.2)	1335	(98.2)	1.00		
> 0 to ≤10	29	(1.6)	13	(1.0)	1.60	(0.80,	3.21)
> 10	21	(1.2)	11	(0.8)	1.90	(0.85,	4.23)
p-trend					0.04		
Frequency of exposure							
Unexposed	1191	(68.8)	910	(71.1)	1.00		
Low: 5 %	76	(4.4)	60	(4.7)	1.21	(0.80,	1.82)
Medium: 6–30 %	351	(20.3)	229	(17.9)	1.10	(0.88,	1.38)
High: >30 %	113	(6.5)	81	(6.3)	1.06	(0.76,	1.48)
p-trend					0.37		
Cumulative occupational exposure^c^							
Unexposed	1133	(64.5)	869	(64.6)	1.00		
Lowest tertile	174	(9.9)	139	(10.3)	0.86	(0.65,	1.14)
Middle tertile	246	(14.0)	183	(13.6)	0.85	(0.66,	1.09)
Highest tertile	158	(11.6)	158	(11.7)	0.89	(0.69,	1.15)
p-trend					0.24		
Total	1771	(100.0)	1360	(100.0)			

^a^Exposures were restricted to estimates with reliability > possible; estimates with low reliability were classified as unexposed

^b^Adjusted for age, province of residence, use of proxy respondents, BMI categories, percent change in weight from maximum lifetime weight, cigarette pack years, combined physical activity index (hours/month), alcohol consumption (servings/week), consumption of processed meat (servings/week), occupational exposure to asbestos (yes/no), occupational exposure to aromatic amines (yes/no)

^c^Cumulative metric of exposure to diesel emissions was derived from estimates of concentration of exposure, frequency of exposure and duration of employment

**Table 3:** Adjusted odds ratios (OR) and corresponding 95 % confidence intervals (CI) for colorectal cancer in relation to occupational exposure to gasoline emissions

Exposure metric^a^	Cases (%)		Controls (%)		OR^b^ (95 % CI)		
Ever exposed							
No	957	(54.0)	783	(42.4)	1.00		
Yes	814	(46.0)	577	(57.6)	1.10	(0.93,	1.31)
Highest attained exposure concentration							
Unexposed	957	(54.0)	783	(57.7)	1.00		
Low	647	(36.5)	462	(34.0)	1.17	(0.97,	1.41)
Medium	109	(6.2)	71	(5.2)	1.04	(0.73,	1.49)
High	58	(3.3)	44	(3.2)	1.04	(0.67,	1.63)
p-trend					0.77		
Duration of exposure (years)							
Unexposed	957	(54.7)	783	(58.1)	1.00		
> 0 to <7	245	(14.0)	179	(13.3)	1.07	(0.84,	1.36)
≥ 7 to ≤26	285	(16.3)	196	(14.5)	1.11	(0.88,	1.40)
> 26	264	(15.1)	190	(14.1)	1.11	(0.84,	1.45)
p-trend					0.32		
Duration of exposure at high concentration (years)							
Unexposed	1713	(96.9)	1316	(97.1)	1.00		
> 0 to ≤5	30	(1.7)	21	(1.6)	0.95	(0.51,	1.75)
> 5	25	(1.4)	18	(1.3)	1.11	(0.57,	2.17)
p-trend					0.83		
Frequency of exposure							
Unexposed	1110	(64.1)	852	(66.6)	1.00		
Low: 5 %	46	(2.7)	56	(4.4)	0.68	(0.43,	1.07)
Medium: 6–30 %	437	(25.3)	282	(22.0)	1.10	(0.89,	1.36)
High: >30 %	138	(8.0)	90	(7.0)	1.09	(0.79,	1.49)
p-trend					0.33		
Cumulative occupational exposure^c^							
Unexposed	957	(54.6)	783	(58.0)	1.00		
Lowest tertile	225	(12.8)	175	(13.0)	0.95	(0.73,	1.24)
Middle tertile	308	(17.6)	204	(15.1)	1.16	(0.93,	1.45)
Highest tertile	262	(15.0)	187	(13.9)	1.13	(0.88,	1.45)
p-trend					0.27		
Total	1771	(100.0)	1360	(100.0)			

^a^Exposures were restricted to estimates with reliability > possible; estimates with low reliability were classified as unexposed

^b^Adjusted for age, province of residence, use of proxy respondents, BMI categories, percent change in weight from maximum lifetime weight, cigarette pack years, combined physical activity index (hours/month), alcohol consumption (servings/week), consumption of processed meat (servings/week), occupational exposure to asbestos (yes/no), occupational exposure to aromatic amines (yes/no)

^c^Cumulative metric of exposure to diesel emissions was derived from estimates of concentration of exposure, frequency of exposure and duration of employment

**Table 4:** Adjusted odds ratios (OR) and corresponding 95 % confidence intervals (CI) for rectal cancer and colon cancer in relation to occupational exposure to diesel emissions

	Rectal cancer (*n* = 840)							Colon cancer (*n* = 931)						
Exposure metric^a^	Cases (%)		Controls (%)		OR^b^	(95 % CI)		Cases (%)		Controls (%)		OR^b^	(95 % CI)	
Ever exposed														
No	538	(64.0)	869	(63.9)	1.00			595	(63.9)	869	(63.9)	1.00		
Yes	302	(36.0)	491	(36.1)	0.89	(0.72,	1.10)	336	(36.1)	491	(36.1)	0.90	(0.73,	1.11)
Highest attained exposure concentration														
Unexposed	538	(64.1)	869	(63.9)	1.00			595	(63.9)	869	(63.9)	1.00		
Low	212	(25.2)	377	(27.7)	0.78	(0.61,	0.99)	238	(25.6)	377	(27.7)	0.81	(0.64,	1.03)
Medium	61	(7.3)	89	(6.5)	0.99	(0.68,	1.44)	75	(8.1)	89	(6.5)	1.09	(0.76,	1.56)
High	29	(3.5)	25	(1.8)	1.98	(1.09,	3.60)	23	(2.5)	25	(1.8)	1.35	(0.72,	2.54)
p-trend	0.56									p-trend		0.91		
Duration of exposure (years)														
Unexposed	538	(64.5)	869	(64.4)	1.00			595	(64.5)	869	(64.4)	1.00		
> 0 to <11	99	(11.9)	157	(11.6)	0.91	(0.67,	1.24)	109	(11.8)	157	(11.6)	0.91	(0.68,	1.24)
≥ 11 to ≤31	112	(13.4)	166	(12.3)	0.99	(0.73,	1.35)	108	(11.7)	166	(12.3)	0.87	(0.64,	1.18)
> 31	85	(10.2)	157	(11.6)	0.76	(0.52,	1.10)	110	(11.9)	157	(11.6)	0.86	(0.60,	1.21)
p-trend	0.16									p-trend		0.31		
Duration of exposure at high concentration (years)														
Unexposed	811	(96.7)	1335	(98.2)	1.00			908	(97.6)	1335	(98.2)	1.00		
> 0 to ≤10	16	(1.9)	13	(1.0)	1.84	(0.84,	4.05)	13	(1.4)	13	(1.0)	1.37	(0.60,	3.14)
> 10	12	(1.4)	11	(0.8)	2.33	(0.94,	5.78)	9	(1.0)	11	(0.8)	1.34	(0.51,	3.55)
p-trend	0.02									p-trend		0.39		
Frequency of exposure														
Unexposed	558	(68.2)	910	(71.1)	1.00			633	(69.3)	910	(71.1)	1.00		
Low: 5 %	33	(4.0)	60	(4.7)	1.27	(0.76,	2.12)	43	(4.7)	60	(4.7)	1.21	(0.75,	1.94)
Medium: 6–30 %	171	(20.9)	229	(17.9)	1.15	(0.87,	1.51)	180	(19.7)	229	(17.9)	1.10	(0.84,	1.44)
High: >30 %	56	(6.9)	81	(6.3)	1.20	(0.80,	1.79)	57	(6.2)	81	(6.3)	0.95	(0.64,	1.41)
p-trend	0.16									p-trend		0.70		
Cumulative occupational exposure^c^														
Unexposed	538	(64.5)	869	(64.4)	1.00			595	(64.5)	869	(64.4)	1.00		
Lowest tertile	87	(10.4)	139	(10.3)	0.87	(0.63,	1.20)	87	(9.4)	139	(10.3)	0.85	(0.61,	1.18)
Middle tertile	116	(13.9)	183	(13.6)	0.88	(0.65,	1.18)	130	(14.1)	183	(13.6)	0.83	(0.63,	1.11)
Highest tertile	93	(11.2)	158	(11.7)	0.92	(0.66,	1.28)	111	(12.0)	158	(11.7)	0.98	(0.71,	1.35)
p-trend	0.38									p-trend		0.42		

^a^Exposures were restricted to estimates with reliability > possible; estimates with low reliability were classified as unexposed

^b^Adjusted for age, province of residence, use of proxy respondents, BMI categories, percent change in weight from maximum lifetime weight, cigarette pack years, combined physical activity index (hours/month), alcohol consumption (servings/week), consumption of processed meat (servings/week), occupational exposure to asbestos (yes/no), occupational exposure to aromatic amines (yes/no)

^c^Cumulative metric of exposure to diesel emissions was derived from estimates of concentration of exposure, frequency of exposure and duration of employment

**Table 5:** Adjusted odds ratios (OR) and corresponding 95 % confidence intervals (CI) for rectal cancer and colon cancer in relation to occupational exposure to gasoline emissions

	Rectal cancer (*n* = 840)							Colon cancer (*n* = 931)						
Exposure metric^a^	Cases (%)		Controls (%)		OR^b^	(95 % CI)		Cases (%)		Controls (%)		OR^b^	(95 % CI)	
Ever exposed														
No	452	(53.8)	783	(57.6)	1.00			505	(54.2)	783	(57.6)	1.00		
Yes	388	(46.2)	577	(42.4)	1.12	(0.91,	1.38)	426	(45.8)	577	(42.4)	1.04	(0.85,	1.27)
Highest attained exposure concentration														
Unexposed	452	(53.8)	783	(57.6)	1.00			505	(54.2)	783	(57.6)	1.00		
Low	305	(36.3)	462	(34.0)	1.20	(0.96,	1.50)	342	(36.7)	462	(34)	1.10	(0.88,	1.37)
Medium	55	(6.6)	71	(5.2)	1.12	(0.73,	1.73)	54	(5.8)	71	(5.2)	0.92	(0.60,	1.42)
High	28	(3.3)	44	(3.2)	1.04	(0.60,	1.79)	30	(3.2)	44	(3.2)	1.04	(0.61,	1.76)
p-trend	0.74										p-trend	0.91		
Duration of exposure (years)														
Unexposed	452	(54.3)	783	(58.1)	1.00			505	(55.0)	783	(58.1)	1.00		
> 0 to <7	117	(14.1)	179	(13.3)	1.09	(0.82,	1.46)	128	(13.9)	161	(11.9)	1.00	(0.76,	1.34)
≥ 7 to ≤26	144	(17.3)	196	(14.5)	1.20	(0.91,	1.59)	141	(15.4)	185	(13.7)	0.96	(0.73,	1.27)
> 26	120	(14.4)	190	(14.1)	1.08	(0.77,	1.51)	144	(15.7)	219	(16.3)	1.10	(0.79,	1.51)
p-trend	0.33										p-trend	0.77		
Duration of exposure at high concentration (years)														
Unexposed	812	(96.8)	1316	(97.1)	1.00			901	(97.0)	1316	(97.1)	1.00		
> 0 to <5	15	(1.8)	21	(1.6)	1.02	(0.48,	2.16)	15	(1.6)	21	(1.6)	0.94	(0.46,	1.94)
≥ 5	12	(1.4)	18	(1.3)	1.08	(0.48,	2.39)	13	(1.4)	18	(1.3)	1.13	(0.51,	2.52)
p-trend	0.84										p-trend	0.84		
Frequency of exposure														
Unexposed	528	(64.6)	852	(66.6)	1.00			582	(63.8)	852	(66.6)	1.00		
Low: 5 %	14	(1.7)	56	(4.4)	0.46	(0.24,	0.89)	32	(3.5)	56	(4.4)	0.85	(0.51,	1.42)
Medium: 6–30 %	213	(26.0)	282	(22.0)	1.08	(0.84,	1.40)	224	(24.5)	282	(22.0)	1.08	(0.84,	1.38)
High: >30 %	63	(7.7)	90	(7.0)	1.00	(0.68,	1.48)	75	(8.2)	90	(7.0)	1.16	(0.80,	1.66)
p-trend	0.72										p-trend	0.30		
Cumulative occupational exposure^c^														
Unexposed	452	(54.3)	783	(58.0)	1.00			505	(55.0)	783	(58.0)	1.00		
Lowest tertile	117	(14.1)	175	(13.0)	1.13	(0.84,	1.51)	108	(11.8)	144	(10.7)	0.85	(0.63,	1.15)
Middle tertile	140	(16.8)	204	(15.1)	1.14	(0.86,	1.51)	168	(18.3)	217	(16.1)	1.13	(0.87,	1.48)
Highest tertile	124	(14.9)	187	(13.9)	1.11	(0.81,	1.51)	138	(15.0)	205	(15.2)	1.06	(0.78,	1.43)
p-trend	0.38										p-trend	0.55		

^a^Exposures were restricted to estimates with reliability > possible; estimates with low reliability were classified as unexposed

^b^Adjusted for age, province of residence, use of proxy respondents, BMI categories, percent change in weight from maximum lifetime weight, cigarette pack years, combined physical activity index (hours/month), alcohol consumption (servings/week), consumption of processed meat (servings/week), occupational exposure to asbestos (yes/no), occupational exposure to aromatic amines (yes/no)

^c^Cumulative metric of exposure to diesel emissions was derived from estimates of concentration of exposure, frequency of exposure and duration of employment

A positive association with CRC was observed for individuals who had been exposed to diesel emissions at high levels of concentration compared to unexposed individuals (OR = 1.65, 95 % CI = 0.98–2.80; p-trend = 0.78). Examining colon and rectal cancer separately (Table 4) revealed that the highest attained concentration of diesel emissions was more strongly associated with rectal (OR = 1.98, 95 % CI = 1.09–3.60; p-trend = 0.56) than colon cancer (OR = 1.35, 95 % CI = 0.72–2.54; p-trend = 0.91). The tests for trend did not reach statistical significance, which suggests that the increase in rectal cancer risk is not linear, and appears to sharply rise only for exposure to high concentrations of diesel emissions.

Duration of exposure to diesel emissions did not appear to be statistically significantly associated with any cancer. However, statistically significant positive trends in cancer risk where revealed when analyses were restricted duration of exposure at high concentrations only. Compared to the unexposed, men with >10 years of diesel exposure at high concentrations had an elevated risk of colorectal (OR = 1.90, 95 % CI = 0.85–4.23; p-trend = 0.02) and rectal cancer (OR = 2.33, 95 % CI = 0.94–5.78; p-trend = 0.02) compared to the unexposed.

Exposure to gasoline emissions was not associated with a marked increase in the risk of CRC, colon or rectal cancers for any of the metrics tested. Notably, cumulative exposure to either diesel or gasoline emissions was not associated with an appreciable increase in the odds of CRC, colon, or rectal cancer.

## Discussion

Exposure to diesel and gasoline emissions is widespread, and accurately assessing its impact on multiple health outcomes continues to be a public health priority. In this study, our aim was to conduct a comprehensive investigation of occupational exposure to diesel and gasoline emissions on colon and rectal cancer risk, using data from a large population-based case–control study. Despite the robust associations previously observed for lung cancer in these data [22] and other studies, the increases in colon and rectal cancer risk appeared to be attenuated. Statistically significant excesses in rectal cancer risk were observed for exposure to diesel emissions at high levels of concentration, as well as for long durations of exposure at high levels of concentration. Exposure to gasoline emissions was not associated with elevated cancer risks in our data.

An important finding of this analysis was the 2-fold increase in the odds of rectal cancer with exposure to diesel exhaust. However, the association observed for the highest attained concentration of diesel exposure and duration of exposure at high concentration should be viewed in the context of weak and null associations observed for other exposure metrics. The lack of monotonically increasing trends in risk for frequency and duration of exposure, suggests that diesel emissions may be associated with rectal cancer risk only in specific occupational sub-groups with prolonged (>10 years) exposure at very high concentrations, such as underground miners, which represent the benchmark for high concentration of diesel exposure in the NECSS. Our results point to concentration as a key dimension of exposure influencing the association with cancer risk. However, given the inconsistent overall pattern of results and the modest prior evidence for a causal relationship, the observed increases in rectal cancer risk should be viewed with caution, and it is possible that some of these associations may be due to chance.

The hypothesis linking diesel emissions to increased risk of colon and rectal cancer postulates that clearance of inhaled the gases and particulate matter by mucocilliary transport and diffusion into the pulmonary capillaries is likely the pathway by which these particles translocate to other organs [32]. Although the carcinogenic mechanism of PAHs has been attributed to genotoxic effects, such as the formation of bulky DNA adducts [33–35], the underlying process is less clear for other constituents of motor vehicle exhaust, such as elemental carbon [35, 36]. Toxicology data suggest that generation of reactive oxygen species (ROS) and the resulting mutations represent another potential carcinogenic mechanism. Dietary exposure to diesel exhaust particles has been linked with ROS-mediated protein oxidation, DNA breaks, and increased formation of DNA-adducts in colon mucosa cells [35]. Studies in experimental models also indicate that a large single dose of exposure to diesel exhaust leads to more pronounced DNA damage, compared to the effects of the same total dose administered in smaller increments over time [37]. These observations suggest that an exposure threshold may exist for the genotoxic effects of diesel exhaust particles, and this is consistent with the results of our analysis, where concentration of exposure, but not duration alone, was associated with cancer risk.

Previous epidemiological studies of diesel and gasoline emissions and CRC risk have reported a somewhat different pattern of results to the one we observed in our data. In a large Canadian population-based case–control study in the 1980’s, exposure to diesel engine exhaust was linked to increased risks of colon cancer (OR = 1.7 for long-term, high-level exposure) [12]. Contrary to our findings, gasoline engine exhaust was associated with elevated risks of rectal cancer (OR = 1.6 for long-term, high-level exposure) [12]. However, this analysis was based on small case series (364 colon and 190 rectal cancers), and lacked information on BMI, diet and physical activity. In another analysis of the same dataset, Goldberg et al. [13] assessed the association of colon cancer with diesel engine emissions in 497 male cases compared with 1514 other cancer patients (excluding cancers of the lung, peritoneum, esophagus, stomach and rectum) and 533 population controls. When the pooled control group was used, the resulting OR for colon cancer was 1.6, whereas the risk increased to 2.1 when only the population-based controls were used [13]. It is plausible that the levels of exposure experienced by population-based controls in our study were higher, which would attenuate the observed associations.

In interpreting the results of our study, it is important to consider that the workplace exposure of NECSS subjects may have been lower than what may be required to produce a detectable increase in cancer risk. The effects of exposure could also be modified by individual differences in ability to metabolize and clear PAHs, and repair DNA damage resulting from oxidative stress generated by other constituents of motor vehicle exhaust [38]. An additional challenge in estimating the effects of diesel and gasoline emissions lies in disentangling the effects of one of these exposures from the other, while also controlling for their mutual confounding. It should also be noted that even if a causal relationship with exposure to diesel emissions exists, it’s contribution to overall CRC risk may be overshadowed by other lifestyle and genetic risk factors. One of the key strengths of our study was the availability of extensive information on anthropometric measures, diet, and physical activity, which allowed us to adjust for these important determinants of CRC risk in a more comprehensive way, compared to previous studies.

Another key advantage of our study is our rigorous and extensive exposure assessment. Compared to studies of job title or industry alone, the expert review enhances our ability to take into consideration idiosyncrasies within each job that can influence exposure dimensions. Experts could take into consideration the time-varying nature of motor vehicle exhaust emissions, resulting from changes in engine technology, use of protective equipment, and policy regulations [39]. The resulting semi-quantitative indices have been shown to be a credible way of assessing exposure, and given the blinded nature of our assessment, this also serves to mitigate the potential for recall bias that is often introduced in self-reported case–control data [40]. Furthermore, our exposure assessment provided information on other occupational agents, such as aromatic amines, asbestos and crystalline silica, which have been taken into account in this analysis.

Despite its many advantages, our exposure assessment also has some limitations. The response rates were modest for cases and controls in our study. Although selection bias resulting from the lower response rate in controls may be a concern, we do not believe that the magnitude of this bias would be large enough to lead to seriously distort the odds ratios for diesel exposure that have been reported. Firstly, it is unlikely that differences in study participation were directly related to diesel and gasoline exposure, since this was not explicitly identified as a study hypothesis during participant recruitment. The potential for selection bias is further reduced since only 1.2 % (*n* = 199) of eligible jobs were excluded from the exposure assessment due to insufficient information.

The impact of non-participation bias is also likely to be minimal because adjustment for socioeconomic factors that are expected to be related to participation, such as education, do not have an appreciable effect on the observed associations with diesel and gasoline exposure. For instance, after adjusting for total years of schooling, the rectal cancer association with exposure to the highest concentration level of diesel emissions remained statistically significant and comparable in magnitude (OR = 1.90 vs. OR = 1.98). A similar pattern was observed for duration of exposure at high concentrations, and other metrics of diesel and gasoline exposure.

Lastly, the associations observed for known colon and rectal cancer risk factors in earlier NECSS publications [41, 42] and in our data, including BMI and dietary factors (i.e.: red and processed meat consumption), also exhibited dose–response patterns that were comparable in magnitude and direction to those that have been previously reported in the literature [7, 9, 43].

The reliance on self-reported job histories makes our data vulnerable to measurement error resulting from inaccuracies in recall, which can decrease statistical power and attenuate the observed associations. Misclassification of exposure likely occurred, although this would have been non-differential, thereby attenuating the observed associations. Moreover, in the absence of direct measurements of exposure, our semi-quantitative indices cannot be used for quantitative risk assessment.

## Conclusions

Our findings are suggestive of a modest association between occupational exposure to high concentrations of diesel emissions and risk of rectal cancer.

## Additional files

Additional file 1: Table S1. Minimally adjusted odds ratios (OR) and corresponding 95 % confidence intervals (CI) for colorectal cancer in relation to occupational exposure to diesel emissions. (DOCX 24 kb) Additional file 2: Table S2. Minimally adjusted odds ratios (OR) and corresponding 95 % confidence intervals (CI) for colorectal cancer in relation to occupational exposure to gasoline emissions. (DOCX 24 kb) Additional file 3: Table S3. Minimally adjusted odds ratios (OR) and corresponding 95 % confidence intervals (CI) for rectal cancer and colon cancer in relation to occupational exposure to diesel emissions. (DOCX 30 kb) Additional file 4: Table S4. Minimally adjusted odds ratios (OR) and corresponding 95 % confidence intervals (CI) for rectal cancer and colon cancer in relation to occupational exposure to gasoline emissions. (DOCX 29 kb)

## Abbreviations

CRC: colorectal cancer
OR: odds ratio
CI: confidence intervals
IARC: International Agency for Research on Cancer
NECSS: National Enhanced Cancer Surveillance System
