# Supplementary material for: Workplace exposure to diesel and gasoline engine exhausts and the risk of colorectal cancer in Canadian men
Source: Environ Health. 2016 Jan 14;15:4. doi: 10.1186/s12940-016-0088-1 (PMC4712563; doi:10.1186/s12940-016-0088-1)
Supplement: Additional file 1: Table S1. — Minimally adjusted odds ratios (OR) and corresponding 95 % confidence intervals (CI) for colorectal cancer in relation to occupational exposure to diesel emissions. (DOCX 24 kb) [file 12940_2016_88_MOESM1_ESM.docx]

**Table S1:** Minimally adjusted odds ratios (OR) and corresponding 95% confidence intervals (CI) for colorectal cancer in relation to occupational exposure to diesel emissions

| **Exposure Metric^1^** | **Cases (%)** | | **Controls (%)** | | **OR^2^ (95% CI)** | | |
| --- | --- | --- | --- | --- | --- | --- | --- |
| Ever exposed |  |  |  |  |  |  |  |
| No | 1133 | (64.0) | 869 | (63.9) | 1.00 |  |  |
| Yes | 638 | (36.0) | 491 | (36.1) | 0.90 | (0.76, | 1.07) |
| Highest attained exposure concentration |  |  |  |  |  |  |  |
| Unexposed | 1133 | (64.0) | 869 | (63.9) | 1.00 |  |  |
| Low | 450 | (25.4) | 377 | (27.7) | 0.79 | (0.65, | 0.96) |
| Medium | 136 | (7.7) | 89 | (6.5) | 1.05 | (0.78, | 1.42) |
| High | 52 | (2.9) | 25 | (1.8) | 1.74 | (1.04, | 2.88) |
| Duration of exposure (years) |  |  |  |  |  |  |  |
| Unexposed | 1133 | (64.5) | 869 | (64.4) | 1.00 |  |  |
| >0 to <11 | 208 | (11.9) | 157 | (11.6) | 0.93 | (0.72, | 1.19) |
| ≥11 to ≤31 | 220 | (12.5) | 166 | (12.3) | 0.94 | (0.73, | 1.20) |
| >31 | 195 | (11.1) | 157 | (11.6) | 0.80 | (0.62, | 1.06) |
| Duration of exposure at high concentration (years) |  |  |  |  |  |  |  |
| Unexposed | 1719 | (97.2) | 1335 | (98.2) | 1.00 |  |  |
| >0 to ≤10 | 29 | (1.6) | 13 | (1.0) | 1.62 | (0.82, | 3.20) |
| >10 | 21 | (1.2) | 11 | (0.8) | 2.06 | (0.96, | 4.42) |
| Frequency of exposure |  |  |  |  |  |  |  |
| Unexposed | 1191 | (68.8) | 910 | (71.1) | 1.00 |  |  |
| Low: 5% | 76 | (4.4) | 60 | (4.7) | 1.23 | (0.83, | 1.83) |
| Medium: 6-30% | 351 | (20.3) | 229 | (17.9) | 1.09 | (0.88, | 1.36) |
| High: >30% | 113 | (6.5) | 81 | (6.3) | 1.18 | (0.85, | 1.63) |
| Cumulative occupational exposure^3^ |  |  |  |  |  |  |  |
| Unexposed | 1133 | (64.5) | 869 | (64.6) | 1.00 |  |  |
| Lowest tertile | 174 | (9.9) | 139 | (10.3) | 0.84 | (0.64, | 1.09) |
| Middle tertile | 246 | (14.0) | 183 | (13.6) | 0.89 | (0.71, | 1.13) |
| Highest tertile | 158 | (11.6) | 158 | (11.7) | 0.93 | (0.71, | 1.20) |
| Total | 1771 | (100.0) | 1360 | (100.0) |  |  |  |

^1^ Exposures were restricted to estimates with reliability > possible; estimates with low reliability were classified as unexposed

^2^ Adjusted for age, province of residence, use of proxy respondents

^3^ Cumulative metric of exposure to diesel emissions was derived from estimates of concentration of exposure, frequency of exposure and duration of employment
