# Supplementary material for: Workplace exposure to diesel and gasoline engine exhausts and the risk of colorectal cancer in Canadian men
Source: Environ Health. 2016 Jan 14;15:4. doi: 10.1186/s12940-016-0088-1 (PMC4712563; doi:10.1186/s12940-016-0088-1)
Supplement: Additional file 2: Table S2. — Minimally adjusted odds ratios (OR) and corresponding 95 % confidence intervals (CI) for colorectal cancer in relation to occupational exposure to gasoline emissions. (DOCX 24 kb) [file 12940_2016_88_MOESM2_ESM.docx]

**Table S2:** Minimally adjusted odds ratios (OR) and corresponding 95% confidence intervals (CI) for colorectal cancer in relation to occupational exposure to gasoline emissions

| **Exposure Metric^1^** | **Cases (%)** | | **Controls (%)** | | **OR^2^ (95% CI)** | | |
| --- | --- | --- | --- | --- | --- | --- | --- |
| Ever exposed |  |  |  |  |  |  |  |
| No | 957 | (54.0) | 783 | (42.4) | 1.00 |  |  |
| Yes | 814 | (46.0) | 577 | (57.6) | 1.16 | (0.98, | 1.36) |
| Highest attained exposure concentration |  |  |  |  |  |  |  |
| Unexposed | 957 | (54.0) | 783 | (57.7) | 1.00 |  |  |
| Low | 647 | (36.5) | 462 | (34.0) | 1.21 | (1.01, | 1.45) |
| Medium | 109 | (6.2) | 71 | (5.2) | 1.23 | (0.88, | 1.72) |
| High | 58 | (3.3) | 44 | (3.2) | 1.13 | (0.74, | 1.74) |
| Duration of exposure (years) |  | |  | |  |  |  |
| Unexposed | 957 | (54.7) | 783 | (58.1) | 1.00 |  |  |
| >0 to <7 | 245 | (14.0) | 179 | (13.3) | 1.13 | (0.89, | 1.42) |
| ≥7 to ≤26 | 285 | (16.3) | 196 | (14.5) | 1.16 | (0.91, | 1.48) |
| >26 | 264 | (15.1) | 190 | (14.1) | 1.16 | (0.90, | 1.48) |
| Duration of exposure at high concentration (years) |  |  |  |  |  |  |  |
| Unexposed | 1713 | (96.9) | 1316 | (97.1) | 1.00 |  |  |
| >0 to ≤5 | 30 | (1.7) | 21 | (1.6) | 1.06 | (0.59, | 1.90) |
| >5 | 25 | (1.4) | 18 | (1.3) | 1.12 | (0.59, | 2.13) |
| Frequency of exposure |  |  |  |  |  |  |  |
| Unexposed | 1110 | (64.1) | 852 | (66.6) | 1.00 |  |  |
| Low: 5% | 46 | (2.7) | 56 | (4.4) | 0.66 | (0.42, | 1.02) |
| Medium: 6-30% | 437 | (25.3) | 282 | (22.0) | 1.10 | (0.90, | 1.35) |
| High: >30% | 138 | (8.0) | 90 | (7.0) | 1.14 | (0.84, | 1.55) |
| Cumulative occupational exposure^3^ |  |  |  |  |  |  |  |
| Unexposed | 957 | (54.6) | 783 | (58.0) | 1.00 |  |  |
| Lowest tertile | 225 | (12.8) | 175 | (13.0) | 1.07 | (0.84, | 1.35) |
| Middle tertile | 308 | (17.6) | 204 | (15.1) | 1.21 | (0.97, | 1.52) |
| Highest tertile | 262 | (15.0) | 187 | (13.9) | 1.16 | (0.91, | 1.49) |
| Total | 1771 | (100.0) | 1360 | (100.0) |  |  |  |

^1^ Exposures were restricted to estimates with reliability > possible; estimates with low reliability were classified as unexposed

^2^ Adjusted for age, province of residence, use of proxy respondents

^3^ Cumulative metric of exposure to diesel emissions was derived from estimates of concentration of exposure, frequency of exposure and duration of employment
