# Supplementary material for: Workplace exposure to diesel and gasoline engine exhausts and the risk of colorectal cancer in Canadian men
Source: Environ Health. 2016 Jan 14;15:4. doi: 10.1186/s12940-016-0088-1 (PMC4712563; doi:10.1186/s12940-016-0088-1)
Supplement: Additional file 3: Table S3. — Minimally adjusted odds ratios (OR) and corresponding 95 % confidence intervals (CI) for rectal cancer and colon cancer in relation to occupational exposure to diesel emissions. (DOCX 30 kb) [file 12940_2016_88_MOESM3_ESM.docx]

**Table S3:** Minimally adjusted odds ratios (OR) and corresponding 95% confidence intervals (CI) for rectal cancer and colon cancer in relation to occupational exposure to diesel emissions

|  | **Rectal Cancer (n=840)** | | | | | | | **Colon Cancer (n=931)** | | | | | | |
| --- | --- | --- | --- | --- | --- | --- | --- | --- | --- | --- | --- | --- | --- | --- |
| **Exposure Metric^1^** | **Cases (%)** | | **Controls (%)** | | **OR^2^** | **(95% CI)** | | **Cases (%)** | | **Controls (%)** | | **OR^3^** | **(95% CI)** | |
| Ever exposed |  |  |  |  |  |  |  |  |  |  |  |  |  |  |
| No | 538 | (64.0) | 869 | (63.9) | 1.00 |  |  | 595 | (63.9) | 869 | (63.9) | 1.00 |  |  |
| Yes | 302 | (36.0) | 491 | (36.1) | 0.90 | (0.73, | 1.10) | 336 | (36.1) | 491 | (36.1) | 0.92 | (0.75, | 1.13) |
| Highest attained exposure concentration | | | | |  |  |  |  |  |  |  |  |  |  |
| Unexposed | 538 | (64.1) | 869 | (63.9) | 1.00 |  |  | 595 | (63.9) | 869 | (63.9) | 1.00 |  |  |
| Low | 212 | (25.2) | 377 | (27.7) | 0.78 | (0.62, | 0.99) | 238 | (25.6) | 377 | (27.7) | 0.81 | (0.65, | 1.03) |
| Medium | 61 | (7.3) | 89 | (6.5) | 0.99 | (0.69, | 1.43) | 75 | (8.1) | 89 | (6.5) | 1.15 | (0.81, | 1.62) |
| High | 29 | (3.5) | 25 | (1.8) | 2.03 | (1.14, | 3.60) | 23 | (2.5) | 25 | (1.8) | 1.48 | (0.80, | 2.71) |
| Duration of exposure (years) | | | | |  |  |  |  |  |  |  |  |  |  |
| Unexposed | 538 | (64.5) | 869 | (64.4) | 1.00 |  |  | 595 | (64.5) | 869 | (64.4) | 1.00 |  |  |
| >0 to <11 | 99 | (11.9) | 157 | (11.6) | 0.92 | (0.68, | 1.24) | 109 | (11.8) | 157 | (11.6) | 0.96 | (0.72, | 1.28) |
| ≥11 to ≤31 | 112 | (13.4) | 166 | (12.3) | 1.00 | (0.74, | 1.34) | 108 | (11.7) | 166 | (12.3) | 0.90 | (0.67, | 1.22) |
| >31 | 85 | (10.2) | 157 | (11.6) | 0.74 | (0.52, | 1.05) | 110 | (11.9) | 157 | (11.6) | 0.85 | (0.61, | 1.18) |
| Duration of exposure at high concentration (years) | | | | |  |  |  |  |  |  |  |  |  |  |
| Unexposed | 811 | (96.7) | 1335 | (98.2) | 1.00 |  |  | 908 | (97.6) | 1335 | (98.2) | 1.00 |  |  |
| >0 to ≤10 | 16 | (1.9) | 13 | (1.0) | 1.79 | (0.83, | 3.85) | 13 | (1.4) | 13 | (1.0) | 1.44 | (0.65, | 3.21) |
| >10 | 12 | (1.4) | 11 | (0.8) | 2.52 | (1.06, | 5.96) | 9 | (1.0) | 11 | (0.8) | 1.64 | (0.64, | 4.15) |
| Frequency of exposure | | | | |  |  |  |  |  |  |  |  |  |  |
| Unexposed | 558 | (68.2) | 910 | (71.1) | 1.00 |  |  | 633 | (69.3) | 910 | (71.1) | 1.00 |  |  |
| Low: 5% | 33 | (4.0) | 60 | (4.7) | 1.26 | (0.76, | 2.07) | 43 | (4.7) | 60 | (4.7) | 1.24 | (0.78, | 1.96) |
| Medium: 6-30% | 171 | (20.9) | 229 | (17.9) | 1.15 | (0.88, | 1.49) | 180 | (19.7) | 229 | (17.9) | 1.09 | (0.84, | 1.41) |
| High: >30% | 56 | (6.9) | 81 | (6.3) | 1.29 | (0.87, | 1.91) | 57 | (6.2) | 81 | (6.3) | 1.08 | (0.74, | 1.58) |
| Cumulative occupational exposure^4^ | | | | |  |  |  |  |  |  |  |  |  |  |
| Unexposed | 538 | (64.5) | 869 | (64.4) | 1.00 |  |  | 595 | (64.5) | 869 | (64.4) | 1.00 |  |  |
| Lowest tertile | 87 | (10.4) | 139 | (10.3) | 0.86 | (0.63, | 1.19) | 87 | (9.4) | 139 | (10.3) | 0.85 | (0.62, | 1.17) |
| Middle tertile | 116 | (13.9) | 183 | (13.6) | 0.91 | (0.68, | 1.21) | 130 | (14.1) | 183 | (13.6) | 0.89 | (0.68, | 1.17) |
| Highest tertile | 93 | (11.2) | 158 | (11.7) | 0.89 | (0.64, | 1.24) | 111 | (12.0) | 158 | (11.7) | 0.97 | (0.71, | 1.33) |

^1^ Exposures were restricted to estimates with reliability > possible; estimates with low reliability were classified as unexposed

^2^ Adjusted for age, province of residence, use of proxy respondents

^3^ Cumulative metric of exposure to diesel emissions was derived from estimates of concentration of exposure, frequency of exposure and duration of employment
