# Supplementary material for: Workplace exposure to diesel and gasoline engine exhausts and the risk of colorectal cancer in Canadian men
Source: Environ Health. 2016 Jan 14;15:4. doi: 10.1186/s12940-016-0088-1 (PMC4712563; doi:10.1186/s12940-016-0088-1)
Supplement: Additional file 4: Table S4. — Minimally adjusted odds ratios (OR) and corresponding 95 % confidence intervals (CI) for rectal cancer and colon cancer in relation to occupational exposure to gasoline emissions. (DOCX 29 kb) [file 12940_2016_88_MOESM4_ESM.docx]

**Table S4:** Minimally adjusted odds ratios (OR) and corresponding 95% confidence intervals (CI) for rectal cancer and colon cancer in relation to occupational exposure to gasoline emissions

|  | **Rectal Cancer (n=840)** | | | | | | | **Colon Cancer (n=931)** | | | | | | |
| --- | --- | --- | --- | --- | --- | --- | --- | --- | --- | --- | --- | --- | --- | --- |
| **Exposure Metric^1^** | **Cases (%)** | | **Controls (%)** | | **OR^2^** | **(95% CI)** | | **Cases (%)** | | **Controls (%)** | | **OR^2^** | **(95% CI)** | |
| Ever exposed |  |  |  |  |  |  |  |  |  |  |  |  |  |  |
| No | 452 | (53.8) | 783 | (57.6) | 1.00 |  |  | 505 | (54.2) | 783 | (57.6) | 1.00 |  |  |
| Yes | 388 | (46.2) | 577 | (42.4) | 1.18 | (0.97, | 1.44) | 426 | (45.8) | 577 | (42.4) | 1.11 | (0.91, | 1.35) |
| Highest attained exposure concentration | | | | |  |  |  |  |  |  |  |  |  |  |
| Unexposed | 452 | (53.8) | 783 | (57.6) | 1.00 |  |  | 505 | (54.2) | 783 | (57.6) | 1.00 |  |  |
| Low | 305 | (36.3) | 462 | (34.0) | 1.23 | (0.99, | 1.53) | 342 | (36.7) | 462 | (34) | 1.15 | (0.93, | 1.42) |
| Medium | 55 | (6.6) | 71 | (5.2) | 1.29 | (0.87, | 1.93) | 54 | (5.8) | 71 | (5.2) | 1.13 | (0.76, | 1.68) |
| High | 28 | (3.3) | 44 | (3.2) | 1.19 | (0.71, | 2.00) | 30 | (3.2) | 44 | (3.2) | 1.12 | (0.67, | 1.87) |
| Duration of exposure (years) | | | | |  |  |  |  |  |  |  |  |  |  |
| Unexposed | 452 | (54.3) | 783 | (58.1) | 1.00 |  |  | 505 | (55.0) | 783 | (58.1) | 1.00 |  |  |
| >0 to <7 | 117 | (14.1) | 179 | (13.3) | 1.15 | (0.87, | 1.52) | 128 | (13.9) | 161 | (11.9) | 1.08 | (0.82, | 1.42) |
| ≥7 to ≤26 | 144 | (17.3) | 196 | (14.5) | 1.22 | (0.92, | 1.63) | 141 | (15.4) | 185 | (13.7) | 1.07 | (0.80, | 1.42) |
| >26 | 120 | (14.4) | 190 | (14.1) | 1.20 | (0.89, | 1.63) | 144 | (15.7) | 219 | (16.3) | 1.12 | (0.84, | 1.50) |
| Duration of exposure at high concentration (years) | | | | |  |  |  |  |  |  |  |  |  |  |
| Unexposed | 812 | (96.8) | 1316 | (97.1) | 1.00 |  |  | 901 | (97.0) | 1316 | (97.1) | 1.00 |  |  |
| >0 to >5 | 15 | (1.8) | 21 | (1.6) | 1.14 | (0.56, | 2.32) | 15 | (1.6) | 21 | (1.6) | 1.03 | (0.51, | 2.08) |
| ≥5 | 12 | (1.4) | 18 | (1.3) | 1.18 | (0.55, | 2.55) | 13 | (1.4) | 18 | (1.3) | 1.14 | (0.53, | 2.45) |
| Frequency of exposure | | | | |  |  |  |  |  |  |  |  |  |  |
| Unexposed | 528 | (64.6) | 852 | (66.6) | 1.00 |  |  | 582 | (63.8) | 852 | (66.6) | 1.00 |  |  |
| Low: 5% | 14 | (1.7) | 56 | (4.4) | 0.42 | (0.22, | 0.81) | 32 | (3.5) | 56 | (4.4) | 0.85 | (0.51, | 1.39) |
| Medium: 6-30% | 213 | (26.0) | 282 | (22.0) | 1.10 | (0.86, | 1.41) | 224 | (24.5) | 282 | (22.0) | 1.07 | (0.84, | 1.36) |
| High: >30% | 63 | (7.7) | 90 | (7.0) | 1.05 | (0.72, | 1.53) | 75 | (8.2) | 90 | (7.0) | 1.26 | (0.88, | 1.78) |
| Cumulative occupational exposure^4^ | | | | |  |  |  |  |  |  |  |  |  |  |
| Unexposed | 452 | (54.3) | 783 | (58.0) | 1.00 |  |  | 505 | (55.0) | 783 | (58.0) | 1.00 |  |  |
| Lowest tertile | 117 | (14.1) | 175 | (13.0) | 1.16 | (0.88, | 1.54) | 108 | (11.8) | 144 | (10.7) | 0.93 | (0.70, | 1.25) |
| Middle tertile | 140 | (16.8) | 204 | (15.1) | 1.19 | (0.91, | 1.57) | 168 | (18.3) | 217 | (16.1) | 1.21 | (0.93, | 1.57) |
| Highest tertile | 124 | (14.9) | 187 | (13.9) | 1.19 | (0.88, | 1.62) | 138 | (15.0) | 205 | (15.2) | 1.12 | (0.84, | 1.50) |

^1^ Exposures were restricted to estimates with reliability > possible; estimates with low reliability were classified as unexposed

^2^ Adjusted for age, province of residence, use of proxy respondents

^3^ Cumulative metric of exposure to diesel emissions was derived from estimates of concentration of exposure, frequency of exposure and duration of employment
